# Different clinical significance of FGFR1–4 expression between diffuse-type and intestinal-type gastric cancer

**DOI:** 10.1186/s12957-016-1081-4

**Published:** 2017-01-05

**Authors:** Mikito Inokuchi, Hideaki Murase, Sho Otsuki, Tatsuyuki Kawano, Kazuyuki Kojima

**Affiliations:** 1 Department of Gastrointestinal Surgery, Tokyo Medical and Dental University, 1-5-45, Yushima, Bunkyo, Tokyo, 113-8519 Japan; 2 Department of Minimally Invasive Surgery, Tokyo Medical and Dental University, 1-5-45, Yushima, Bunkyo, Tokyo, 113-8519 Japan

**Keywords:** Fibroblast growth factor receptor 1, Fibroblast growth factor receptor 2, Fibroblast growth factor receptor 3, Fibroblast growth factor receptor 4, Gastric cancer, Immunohistochemistry

## Abstract

**Background:**

Receptor tyrosine kinases promote tumor progression in many cancers, although oncologic activation differs between diffuse-type gastric cancer (DGC) and intestinal-type gastric cancer (IGC). Fibroblast growth factor receptor (FGFR) is one RTK, and we previously reported the clinical significance of FGFR1, 2, 3, and 4 in gastric cancer. The aim of the present study was to reevaluate the clinical significance of FGFR1–4 expression separately in DGC and IGC.

**Methods:**

Tumor samples, including 109 DGCs and 100 IGCs, were obtained from patients who underwent gastrectomy between 2003 and 2007 in our institution. The expression levels of FGFR1, 2, 3, and 4 were measured in the tumors by immunohistochemical analysis.

**Results:**

In DGC, high expression of FGFR1, FGFR2, or FGFR4 was significantly associated with the depth of invasion, lymph-node metastasis, pathological stage, and distant metastasis or recurrent disease. Patients with high expression of FGFR1, FGFR2, or FGFR4 had significantly poorer disease-specific survival (DSS) (*p* = 0.009, *p* = 0.001, and *p* = 0.023, respectively). In IGC, only FGFR4 expression was significantly associated with factors relative to tumor progression and with shorter DSS (*p* = 0.012).

**Conclusion:**

In conclusion, high FGFR4 expression correlated with tumor progression and survival in both DGC and IGC, whereas high expression of FGFR1 and 2 correlated with tumor progression and survival in only DGC.

## Background

Gastric cancer (GC) is categorized into three types based on Lauren’s pathological classification: diffuse type, intestinal type, and mixed type [1]. Diffuse-type gastric cancer (DGC) is associated with more advanced disease stage and poorer survival than intestinal-type gastric cancer (IGC) [2, 3]. It is well known that protein or gene overexpression of receptor tyrosine kinases (RTKs) correlates with tumor progression and poor survival in GC [4, 5]. The immunohistochemical overexpression of human epidermal growth factor receptor 2 (HER2), one of the RTKs, was detected more frequently in IGC than in DGC [6]. Comprehensive genomic analyses performed in The Genomic Cancer Atlas (TGCA) project revealed different genomic alterations of RTKs between DGC and IGC [7]. Therefore, the impact of RTK overexpression on clinical outcomes might differ between DGC and IGC.

The fibroblast growth factor receptor (FGFR) family comprises one type of RTK that regulates fundamental developmental pathways by interacting with fibroblast growth factors (FGFs). FGF signaling participates in several biological functions in the adult organism, including regulation of angiogenesis and wound repair. FGFRs are expressed on a number of different cell types and regulate key cell activities, such as proliferation, survival, migration, and differentiation [8]. *FGFR2* gene amplification was initially found in a GC cell line originating from DGC with the poorest prognosis [9]. Gene amplification or protein overexpression of *FGFR2* has been reported in GC, leading to poor outcomes [10]. Moreover, GC cell lines presenting with *FGFR2* amplification are highly sensitive to inhibition of FGFR signaling by tyrosine kinase inhibitors and monoclonal antibodies in preclinical models [11, 12]. FGFR2 has thus attracted considerable attention as a novel therapeutic candidate for the development of targeted anticancer agents [13].

We previously reported the relations of the immunohistochemical expressions of FGFR1–4 to tumor progression or poor survival in GC. However, tumors were classified into differentiated and undifferentiated types based on the World Health Organization pathological classification in the previous study and were not classified according to Lauren’s classification [14]. The present study was designed to reevaluate the clinical significance of FGFR1–4 expression separately in DGC and IGC diagnosed according to Lauren’s classification, excluding mixed-type GC.

## Methods

### Patients

GC tissue samples were obtained from 222 patients with primary gastric adenocarcinoma who underwent surgical resection between January 2003 and December 2007 in our institution. Each tumor was examined pathologically and classified according to the tumor–node–metastasis staging system recommended by the Union for International Cancer Control (UICC). Of the 222 patients, 109, 100, and 13 tumors were pathologically diagnosed as DGC, IGC, and mixed-type GC, respectively. We excluded the 13 patients with mixed-type GC from the present study. All patients were evaluated for recurrent disease by diagnostic imaging (computed tomography, ultrasonography, magnetic resonance imaging, and endoscopy) every 3 to 6 months. The median follow-up was 61 months (range, 4 to 111 months). HER2 status was evaluated by pathologists in our institution and scored according to standardized assessment criteria [15].

### Immunostaining of the FGFR family

The detailed method used to perform immunohistochemical analysis was described in our previous report [14]. Representative formalin-fixed, paraffin-embedded tissue blocks were sliced into 4-μm-thick sections. After deparaffinization and rehydration, antigen retrieval was performed. Subsequently, endogenous peroxidase and non-specific binding were blocked. The slides were incubated with the primary polyclonal rabbit antibodies, including anti-FGFR1 (dilution, 1:100), anti-FGFR2 (dilution, 1:300), anti-FGFR3 (dilution, 1:500), and anti-FGFR4 (dilution, 1:100), in 1% bovine serum albumin/phosphate-buffered saline overnight at 4 °C. All primary antibodies (named sc-121, 122, 123, and 124 for FGFR1, 2, 3, and 4, respectively) were purchased from Santa Cruz Biotechnology, Inc. (Santa Cruz, CA, USA). The sections were then incubated with secondary antibodies, Histofine Simple Stain MAX PO (Multi) (Nichirei Co., Tokyo, Japan), for 30 min at room temperature. The chromogen substrate was 3,3′-diaminobenzidine tetrahydrochloride solution (Histofine Simple Stain DAB solution, Nichirei Co.). Subsequently, the sections were counterstained with Mayer’s hematoxylin (Wako, Tokyo, Japan). Negative controls were treated similarly, except that the antibodies were replaced by normal rabbit IgG (Santa Cruz Biotechnology, Inc.).

### Interpretation of immunostaining

The assessment of FGFR1–4 staining was based on a previous study of FGFR2 [5], although the criteria were modified slightly and simplified. The staining intensity was scored into three grades as follows: 0, no staining; 1, weakly positive; and 2, moderately or strongly positive. The staining extent (positive frequency) was also scored into three grades according to the percentage of stained tumor cells as follows: 0, <10%; 1, 10% to 50%; and 2, >50% stained cells. For the statistical analysis, composite scores were calculated by addition of the intensity and extent scores. Composite scores of ≥3 were defined as high expression, and scores of <3 as low expression. Two investigators who were blinded to the clinical outcomes separately counted the stained cancer cells. Any disagreements between the two investigators were resolved by reassessment and consensus.

### Statistical analysis

Categorical data were compared with the use of the chi-square test or Fisher’s exact test, as appropriate. Kaplan-Meier curves were plotted to assess the effects of FGFR expression on disease-specific survival (DSS), and different DSS curves were compared using the log-rank test. Multivariate proportional Cox models were used to assess the prognostic significance of FGFR and of factors significantly associated with DSS. Values of *p* < 0.05 were considered to indicate statistical significance. All analyses were performed with the statistical software package SPSS 22 (SPSS Japan Inc., Tokyo, Japan).

## Results

### DGC

**Fig. 1:**
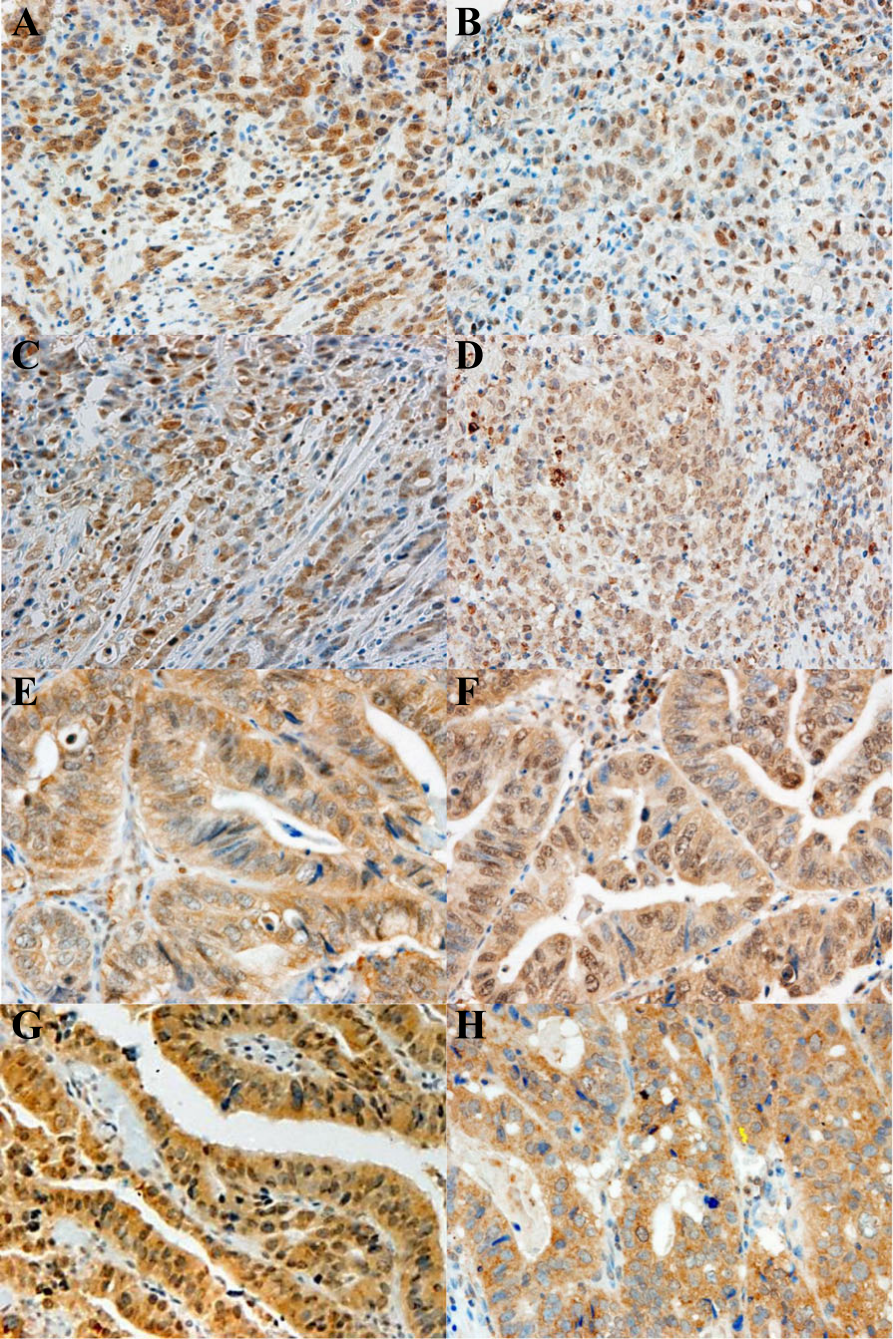
Immunostaining for fibroblast growth factor receptor (FGFR)1, FGFR2, FGFR3, and FGFR4. Representative primary gastric carcinomas exhibiting high expression for (**a**) FGFR1, (**b**) FGFR2, (**c**) FGFR3, and (**d**) FGFR4 in diffuse-type gastric cancer. Representative primary gastric carcinomas exhibiting high expression for (**e**) FGFR1, (**f**) FGFR2, (**g**) FGFR3, and (**h**) FGFR4 in intestinal-type gastric cancer. Magnification, ×400

Among the 109 DGC tumors studied, high expression of FGFR1, 2, 3, and 4 was shown by 40 (37%), 53 (49%), 65 (60%), and 88 (81%) tumors, respectively (Fig. 1). In a previous study, FGFRs were not stained or only weakly stained in normal gastric epithelium [14]. The relations of various clinicopathological factors to the expression levels of FGFR1–4 are summarized in Table 1. High expression levels of FGFR1, 2, and 4 were significantly associated with the depth of invasion (early cancer vs. advanced cancer), lymph-node metastasis (negative vs. positive), tumor stage (stage I vs. stage II or more advanced), and distant metastasis or recurrence (negative vs. positive). Only FGFR3 expression did not correlate with those factors. High FGFR1 expression significantly correlated with peritoneal dissemination, and high FGFR2 expression tended to be associated with peritoneal dissemination. Few DGC tumors were associated with hematogenous metastasis (liver or lung metastasis) or HER2 score.

**Table 1:** Clinicopathological factors and expressions of FGFR1 to FGFR4 in DGC

		FGFR1			FGFR2			FGFR3			FGFR4		
		Low	High	*p*	Low	High	*p*	Low	High	*p*	Low	High	*p*
	*n*	69	40		56	53		44	65		21	88	
Age (years)													
< 65	61	45	16	0.011	37	24	0.029	24	37	0.81	15	46	0.11
≥ 65	48	24	24		19	29		20	28		6	42	
Gender													
Female	40	25	15	0.90	22	18	0.56	11	29	0.037	8	32	0.88
Male	69	44	25		34	35		33	36		13	56	
Main location													
Middle or lower	83	54	29	0.50	46	37	0.13	28	55	0.012	16	67	1.00
Upper	26	15	11		10	16		16	10		5	21	
Depth of invasion													
Early (T1)	35	29	6	0.004	26	9	0.001	18	17	0.11	12	23	0.006
Advanced (T2/3/4)	74	40	34		30	44		26	48		9	65	
LN metastasis													
Negative (N0)	45	35	10	0.009	30	15	0.007	19	26	0.74	16	29	<0.001
Positive (N1/2/3)	64	34	30		26	38		25	39		5	59	
Stage													
I	43	36	7	<0.001	30	13	0.002	21	22	0.15	16	27	<0.001
II/III/IV	66	33	33		26	40		23	43		5	61	
Distant metastasis or recurrence													
Negative	67	50	17	0.002	43	24	0.001	28	39	0.70	18	49	0.01
Positive	42	19	23		13	29		16	26		3	39	
Peritoneal dissemination													
Negative	82	58	24	0.005	46	36	0.086	35	47	0.39	18	64	0.22
Positive	27	11	16		10	17		9	18		3	24	
Hematogenous metastasis													
Negative	106	67	39	1.00	56	50	0.11	42	64	0.56	21	85	1.00
Positive	3	2	1		0	3		2	1		0	3	
HER2 score													
0–1	106	68	38	0.56	55	51	0.61	43	63	1.00	21	85	1.00
2–3	3	1	2		1	2		1	2		0	3	

**Fig. 2:**
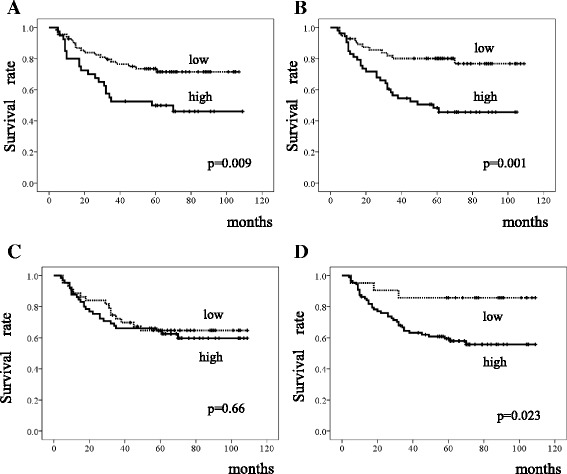
Survival of patients with diffuse-type gastric cancer. Kaplan-Meier curves for the disease-specific survival of patients with expression of (**a**) fibroblast growth factor receptor (FGFR)1, (**b**) FGFR2, (**c**) FGFR3, and (**d**) FGFR4

Patients whose tumors showed high expression of FGFR1, FGFR2, or FGFR4 had significantly poorer DSS on univariate analysis (*p* = 0.009, *p* = 0.001, and *p* = 0.023, respectively; Fig. 2). FGFR3 was not significantly associated with DSS. Median follow-up times did not differ significantly between high and low expression of any FGFR. Cox proportional-hazards regression analysis of DSS was performed, with adjustment for the following clinical variables shown to be prognostic factors on univariate analysis: age (≥65 vs. <65), main location (middle or lower vs. upper), depth of tumor invasion, lymph-node metastasis, and FGFR1, FGFR2, and FGFR4 expression (low vs. high). However, multivariate analysis indicated that expression levels of FGFR1–4 were not significant independent prognostic factors for DSS (Table 2). The depth of invasion (hazard ratio [HR] 8.9, 95% confidence interval [CI] 1.2–68, *p* = 0.034) and lymph-node metastasis (HR 6.1, 95% CI 1.8–21, *p* =​ 0.004) were independent prognostic factors for DSS.

**Table 2:** Prognostic factors for DSS on multivariate analysis in DGC

	Univariate (log-rank)		Multivariate		
	5-yr DSS (%)	*p*	HR	95% CI	*p*
Age (years)					
< 65	73		1		
≥ 65	51	0.013	1.4	0.71-2.6	0.36
Gender					
Female	70				
Male	61	0.52			
Main location					
Middle or lower	72		1		
Upper	41	0.010	1.8	0.90–3.4	0.097
Depth of invasion					
Early (T1)	97		1		
Advanced (T2/3/4)	49	<0.001	8.9	1.2-68	0.034
LN metastasis					
Negative (N0)	95		1		
Positive (N1/2/3)	43	<0.001	6.1	1.8–21	0.004
FGFR1					
Low	74		1		
High	50	0.009	1.2	0.60–2.3	0.66
FGFR2					
Low	80		1		
High	48	0.001	1.5	0.69–3.1	0.32
FGFR3					
Low	65				
High	65	0.66			
FGFR4					
Low	86		1		
High	60	0.023	1.1	0.32–3.7	0.89

### IGC

Among the 100 IGC tumors, high expression of FGFR1, 2, 3, and 4 was shown by 38 (38%), 64 (64%), 76 (76%), and 81 (81%) tumors, respectively (Fig. 1). The relations of clinicopathological variables to the expression levels of FGFR1–4 are summarized in Table 3. Only high FGFR4 expression was significantly associated with the following factors closely related to tumor growth: depth of invasion, lymph-node metastasis, tumor stage, and distant metastasis or recurrence. However, FGFR expression levels did not correlate with the site of recurrence. FGFR1, 2, and 3 were not significantly associated with any clinicopathological variable. HER2 score did not significantly correlate with any FGFR.

**Table 3:** Clinicopathological variables and expressions from FGFR1 to FGFR4 in IGC

		FGFR1			FGFR2			FGFR3			FGFR4		
		Low	High	*p*	Low	High	*p*	Low	High	*p*	Low	High	*p*
	*n*	62	38		36	64		24	76		19	81	
Age (years)													
< 65	32	19	13	0.71	10	22	0.50	7	25	0.73	9	23	0.11
≥ 65	68	43	25		26	42		17	51		10	58	
Gender													
Female	13	5	8	0.073	3	10	0.37	3	10	1.00	1	12	0.45
Male	87	57	30		33	54		21	66		18	69	
Main location													
Middle or lower	84	53	31	0.61	32	52	0.32	19	65	0.53	15	69	0.50
Upper	16	9	7		4	12		5	11		4	12	
Depth of invasion													
Early (T1)	49	34	15	0.14	22	27	0.069	12	37	0.91	17	32	<0.001
Advanced (T2/3/4)	51	28	23		14	37		12	39		2	49	
LN metastasis													
Negative (N0)	66	42	24	0.64	26	40	0.33	16	50	0.94	17	49	0.016
Positive (N1/2/3)	34	20	14		10	24		8	26		2	32	
Stage													
I	60	40	20	0.24	25	35	0.15	15	45	0.77	18	42	0.001
II/III/IV	40	22	18		11	29		9	31		1	39	
Distant metastasis or recurrence													
Negative	80	53	27	0.080	31	49	0.25	17	63	0.24	19	61	0.01
Positive	20	9	11		5	15		7	13		0	20	
Peritoneal dissemination													
Negative	93	59	34	0.42	34	59	1.00	21	72	0.35	19	74	0.34
Positive	7	3	4		2	5		3	4		0	7	
Hematogenous metastasis													
Negative	90	56	34	1.00	34	56	0.32	22	68	1.00	19	71	0.20
Positive	10	6	4		2	8		2	8		0	10	
HER2 score													
0–1	84	50	34	0.24	30	54	0.89	19	65	0.53	18	66	0.29
2–3	16	12	4		6	10		5	11		1	15	

**Fig. 3:**
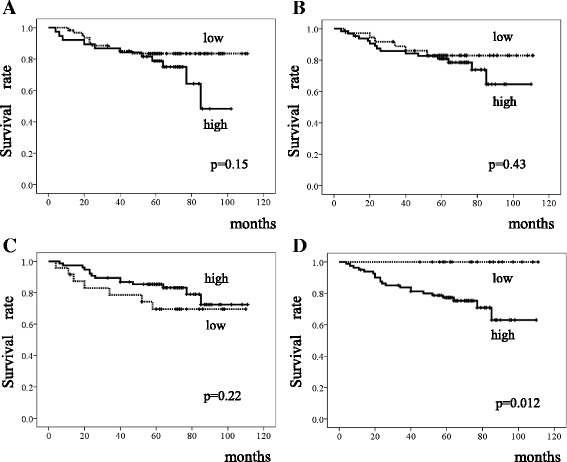
Survival of patients with intestinal-type gastric cancer. Kaplan-Meier curves for the disease-specific survival of patients with expression of (**a**) fibroblast growth factor receptor (FGFR)1, (**b**) FGFR2, (**c**) FGFR3, and (**d**) FGFR4

Patients whose tumors showed high FGFR4 expression had significantly shorter DSS than those with low FGFR4 expression on univariate analysis (*p* = 0.012); expression levels of the other FGFRs did not significantly correlate with DSS (Fig. 3). Median follow-up times did not differ significantly between high and low expression of any FGFR. Multivariate analysis using a Cox regression hazard model could not be performed, because no patient with low expression of FGFR4 died of GC.

## Discussion

Our results suggested that the clinical significance of the immunohistochemical expression of FGFRs might differ between DGC and IGC. High FGFR4 expression was frequently found in DGC and even in IGC and was significantly related to tumor progression and metastasis in both types of GC. Previous studies showed that overexpression of FGFR4 protein or *FGFR4* mRNA correlated with shorter survival in GC [16, 17]. However, FGFR4 protein was not significantly associated with clinicopathological factors such as tumor depth or lymph-node metastasis [16, 18]. FGFR4 protein overexpression was shown to be an independent prognostic factor in non-small cell lung cancer [19] and colorectal cancer [20]. In addition, the FGFR4 Arg388 allele, leading to high FGFR protein expression, correlated with shorter survival in GC [21]. In an in vitro study, FGFR4 showed different intracellular sorting patterns from those of FGFR1–3. FGFR4 and its bound ligand were sorted mainly to the recycling compartment and could prolong signaling, whereas FGFR1, 2, and 3 with their ligands were sorted mainly to degradation in lysosomes [22]. Colorectal cancer cell lines cocultured with tumor-associated fibroblasts (TAF) induced significant overexpression of FGFR4, but not of other FGFRs [23]. In addition, FGFR4 plays crucial roles in TAF-induced epithelial-to-mesenchymal transition [23]. Thus, FGFR4 might play a different role from other FGFRs in malignant tumors.

In the present study, high FGFR2 expression significantly correlated with tumor progression and survival in only DGC, and such expression was likely to be associated with peritoneal dissemination. On the basis of whole-genome sequence data, many IGCs were classified as chromosomally unstable tumors, in which RTK-RAS signal transduction pathway is often activated [7]. Moreover, FGFR2 amplification was mutually exclusive from the amplification of other RTKs [4]. The overexpression of HER2 or c-MET was observed more frequently in IGC than in DGC [6, 24], suggesting that a signaling pathway activated by these RTKs might have a critical role in the progression and prognosis of IGC. HER2 overexpression was often found in IGC without significant association of FGFRs in this study, and our results might support those of another study reporting exclusive RTK expression [5]. In a previous review, FGFR2 protein overexpression on immunohistochemical analysis was found more frequently in undifferentiated GC than in differentiated GC [10]. Another study reported that GC tumors with FGFR2 protein overexpression were significantly more common in DGC than in IGC [25]. A further study showed that FGFR2 protein overexpression was significantly associated with poor survival and peritoneal dissemination in GC [5]. These findings suggest that FGFR2 can contribute to the development of DGC or undifferentiated GC, often in association with peritoneal dissemination. However, FGFR2 overexpression was similarly observed in differentiated GC and undifferentiated GC in a study of 950 Japanese patients [5]. *FGFR2* gene amplification was initially detected in a scirrhous-type GC cell line [26]. Similar presences of *FGFR2* amplification in DGC and IGC or in undifferentiated GC and differentiated GC have been reported by some studies; however, such amplification was not often found in GC (1.8 to 9.3%) [4, 27–32]. *FGFR2* gene amplification was significantly related to poor survival in GC [27–31]. A meta-analysis including various types of cancers showed that *FGFR2* amplification significantly correlated with poor survival [33]. FGFR2 inhibitors are being studied as anticancer agents against *FGFR2*-amplified GC in ongoing clinical trials [10].

High FGFR1 protein expression was significantly associated with poor survival and the presence of peritoneal dissemination in only DGC in the present study. To our knowledge, no previous study has assessed the clinical significance of FGFR1 protein expression in DGC. Amplification of the *FGFR1* gene was a rare but noticeable event that was found in 2% (6 of 293) of GCs and was associated with distant metastasis and poor survival in another study, although tumors with *FGFR1* amplification were found in IGC, DGC, and mixed-type GC [34]. FGFR1 protein expression of ≥1% in tumors was associated with poor survival in patients with breast cancer [35]. *FGFR1* amplification was also associated with poor survival in esophageal cancer [36], breast cancer [37], and squamous-cell lung cancer [38]. In a study of colorectal cancer, the copy number gain of *FGFR1* significantly correlated with worse outcomes [39]. The clinical significance of FGFR3 protein expression differs somewhat among tumor types. FGFR3 protein expression was not associated with any clinicopathological feature in the present study, although few studies of FGFR3 expression in gastrointestinal cancers have been reported. No relation was found between FGFR3 protein expression and clinicopathological features in breast cancer [40]. FGFR3 protein expression did not correlate with survival in urothelial carcinoma of the bladder [41]. In contrast, FGFR3 protein expression was significantly associated with shorter survival in multiple myeloma [42].

In our study, FGFRs were expressed mainly in the cytoplasm and partially even in the nucleus. FGFR2 and 4 were mainly found in the cytoplasm of GC cells in other studies [5, 17], which was supported by our results. Epidermal growth factor receptor (EGFR) or HER2 was expressed mainly in the membrane on immunohistochemical analysis, although other RTKs, such as HER3 or cMET, were found not only in the membrane but also in the cytoplasm or nucleus of GC cells [43, 44]. EGFR was also transported in the nucleus, and nuclear localized EGFR is strongly associated with disease progression and worse overall survival in numerous cancers [45]. The status of *Helicobacter pylori* infection was not investigated in this study. However, infection with CagA-positive strains of *Helicobacter pylori* was significantly associated with the presence of GC in both IGC and DGC [46].

## Conclusion

In conclusion, the protein expressions of FGFR1–4 had different impacts on clinical outcomes in DGC and IGC. High FGFR4 expression correlated with tumor progression and survival in both types of GC, although FGFR1 and 2 correlated with these variables in only DGC. Therefore, FGFR inhibitors might be more effective for DGC than IGC.

## Abbreviations

DGC: Diffuse-type gastric cancer
DSS: Disease-specific survival
FGFR: Fibroblast growth factor receptor
GC: Gastric cancer
IGC: Intestinal-type gastric cancer
RTK: Receptor tyrosine kinase
